# Middle Ear Prosthesis with Bactericidal Efficacy—In Vitro Investigation

**DOI:** 10.3390/molecules22101681

**Published:** 2017-10-10

**Authors:** Magdalena Ziąbka, Michał Dziadek, Elżbieta Menaszek, Rafał Banasiuk, Aleksandra Królicka

**Affiliations:** 1 AGH University of Science and Technology, Faculty of Materials Science and Ceramics, Department of Ceramics and Refractories, Krakow 30-059, Poland; 2 AGH University of Science and Technology, Faculty of Materials Science and Ceramics, Department of Glass Technology and Amorphous Coatings, Krakow 30-059, Poland; dziadek@agh.edu.pl; 3 UJ-Jagiellonian University, Medical College, Faculty of Pharmacy, Department of Cytobiology, Krakow 30-001, Poland; elzbieta.menaszek@uj.edu.pl; 4 University of Gdansk, Intercollegiate Faculty of Biotechnology UG-GUMed, Department of Biotechnology, Laboratory of Biologically Active Compounds, Gdansk 80-307, Poland; rafal.banasiuk@biotech.ug.edu.pl (R.B.); aleksandra.krolicka@biotech.ug.edu.pl (A.K.)

**Keywords:** ossicular reconstruction, middle ear prosthesis, bactericidal efficacy, cytotoxicity

## Abstract

Materials used in ossicular replacement prostheses must possess appropriate biological properties, such as biocompatibility, stability, no cytotoxicity. Due to the risk of infection (otitis media and chronic otitis media), it is desirable to use an antibacterial agent for illness prevention during the ossicular reconstruction. The goal of this work was to observe biological properties of a new composite prosthesis made of ABS containing silver nanoparticles (AgNPs 45T). Samples for biological tests and then a prototype of middle ear prosthesis were prepared using injection moulding and extrusion techniques. In vitro experiments were carried out to assess bactericidal efficacy against *Staphylococcus aureus* and *Pseudomona aeruginosa* standard strains, cell proliferation, viability and cytotoxicity, using Hs680.Tr. fibroblast cells. Surface parameters of the samples were evaluated, including roughness and wettability. The silver ions were continually released from the polymer in aqueous solution. The silver ions release was measured as increasing with time and concentration of the silver nanoparticles in the polymer matrix. No cytotoxicity effect was observed, while bactericidal efficacy was noticed for silver nanoparticles. The roughness studies showed an increase in roughness for the samples with silver nanoparticles. All polymer and composite materials containing silver nanoparticles showed hydrophilic properties. The composites were found to release silver ions at a concentration level capable of rendering the antimicrobial efficacy even with the lowest concentration of silver nanoparticles in the material. Our results demonstrate that middle ear prosthesis made of polymer and silver nanoparticles may eliminate bacteria during inflammation in the middle ear.

## 1. Introduction

Otitis media is one of the most common illnesses in otolaryngology, which might result in loss of hearing due to the ossicular chain damage. There are several types of bacteria which cause otitis media [1], thus the necessity to perform bacteriological diagnosis, especially in situations when conservative treatment does not bring expected results. On the grounds of widespread application of antibiotics and the increasing resistance of microorganisms, there is a need to develop modern methods of dealing with chronic infections [2]. Silver nanoparticles (AgNPs) that exhibit antimicrobial efficacy seem a good alternative [3,4]. Ideal middle ear implants should not only reconstruct the bone structure and recover its functions, but also shorten the recovery period and limit risks related to the bacterial infection. Apart from healing chronic middle ear diseases, modern surgery is supposed to restitute hearing abilities [5,6]. In cases of vast damages it is recommended to use prostheses. Nowadays technology provides an opportunity to design and develop implants of various shapes and sizes using different kinds of materials (metals, ceramics, plastics and composites). There is a variety of materials to choose from when performing ossicular reconstruction, including autogenous tissues and alloplasts. The most commonly used prosthesis for ossicular chain reconstruction is a hydroxyapatite prosthesis. The results indicate that hydroxyapatite is a suitable material for an ossicular prosthesis, although the incidence of extrusion is high when it is placed in contact with the tympanic membrane [7]. Literature data reveals that titanium ossicular prostheses are the most popular among surgeons because they are efficient in sound transmission, delicate and easy to handle [8]. Titanium is the most lightweight and biocompatible material among all the allogenic materials used for ossicular reconstruction [9].

Several alloplastic materials have also been considered applicable for ossicular reconstruction. Polyethylene, high-density polyethylene sponge (HDPS), polytetrafluoroethylene (PTFE), and Proplast (PTFE–carbon composite) pistons are used in middle ear reconstruction [10]. However, these materials demonstrate extrusion, migration, and reactivity. The prostheses made of polymers are easy to handle but the extrusion rate is high when they are placed directly below the eardrum [11]. Apart from metallic, ceramic and polymer prostheses, the tendency is to use hybrid prostheses made of materials that combine good biocompatibility with a lower rate of extrusion [12]. There have been numerous reports on the use of different artificial ossicular materials in recent years, but questions remain with regard to histocompatibility and long-term outcomes [13].

According to the WHO, in 2012 about 360 million people suffered from hearing deterioration, including total loss of hearing sense (5.3% of world population), 328 million (91%) of them are adults, 32 million (9%) are children. In the USA in 2010 a diagnosed number of patients with hearing loss exceeded 30 million. In Poland, in Cracow there are between 150 and 170 surgeries annually (performed in The Department of Otolaryngology, Jagiellonian University in Krakow). These figures prove the necessity to conduct further research on opportunities and solutions in dealing with loss of hearing as one of civilization diseases.

In the surgery of ossicular replacement prosthesis none of implants possess bactericidal properties. Therefore, antimicrobial polymers are highly demanded as a strategy to avoid otitis media infections [14]. Thanks to the silver nanoparticles incorporated in the polymer matrix, prostheses demonstrate bactericidal properties and shorten a recovery period. In our case, the whole prosthesis is made of thermoplastic polymer (ABS), which makes it lightweight and possible to adjust the length. The round shape of the head plate minimizes the risk of tympanic membrane damage. The openwork construction of prosthesis (antenna) allows its easy placement in the middle ear and gives a possibilities to manually form a desired shape, according to the particular ossicular chain damages. Moreover, the mechanical properties, such as Young’s modulus, are similar to the bone. After all, the cheap manufacturing method makes the product competitive in scope of general costs of treatment. The novelty is also the antibacterial function of the plastic prosthesis. This medical device is similar to the titanium prostheses in shape but up to now it has never been manufactured by injection moulding and extrusion.

## 2. Results

**Figure 1:**
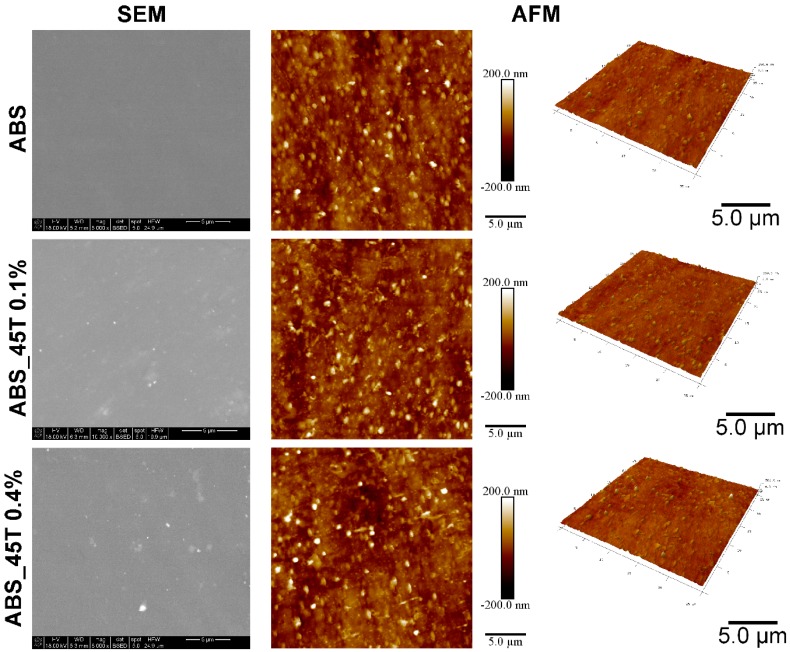
SEM and AFM tapping mode images of pure polymer and polymer containing 0.1% and 0.4% of silver nanoparticles AgNPs 45T by weight.

Based on both SEM and AFM images with the same magnification, silver nanoparticles are homogenously distributed in the polymer matrix (Figure 1). The SEM analysis conducted using BSE detector confirmed the increasing content of the silver nanoparticles, depending on the modifier amount. The silver nanoparticles are observed as a light area on SEM images, which was proved by EDX analysis. In the sample containing a higher amount of nonofillers (ABS_45T 0.4%) some small aggregates were present. Such a behavior may result from to the ability of silver nanoparticles to form aggregates. EDX analysis obtained from 10 measurements for each sample showed a homogenous distribution of elements in the whole investigated area (Table 1). The EDX silver content is compatible with the assumed nanosilver content in the materials. AFM images did not show significant differences in the surface morphology. However, Ra and Rq parameters, obtained from AFM images, indicate statistically significant changes with the increasing content of silver nanoadditives.

**Figure 2:**
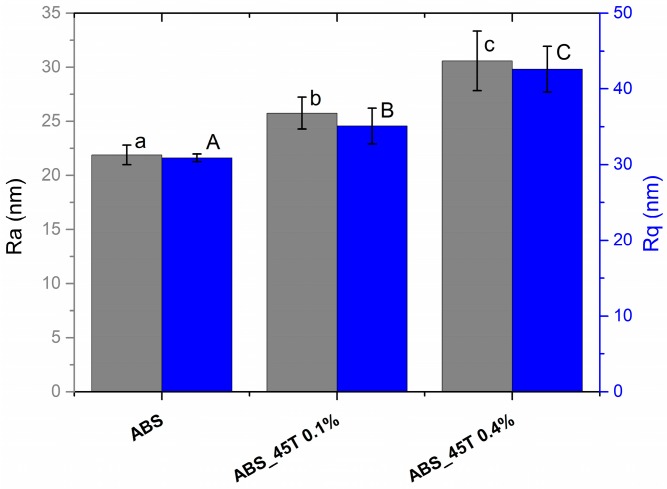
The average roughness (Ra), root mean-square roughness (Rq) of pure polymer and polymer containing 0.1% and 0.4% of silver nanoparticles AgNPs 45T by weight obtained from AFM images. Statistically significant differences (*p* < 0.05) between the tested materials are marked a–c for Ra and A–C for Rq, respectively.

Surface roughness tests using AFM were conducted to establish the topography of the materials and distribution of silver nanoparticles on the samples surface. The basic roughness parameters (Ra, Rq) were obtained (Figure 2). The AFM tests proved that all the materials are characterized by the arithmetic average of the roughness profile (Ra) below 35 nm and root mean-square roughness (Rq) below 50 nm, which indicates a low surface roughness. The roughness increase is connected with the amount of the modifier–statistically, the more nanosilver was incorporated into the polymer matrix, the more nanopowder is observed closer to the surface. Therefore, there are bigger differences in the profile heights.

**Figure 3:**
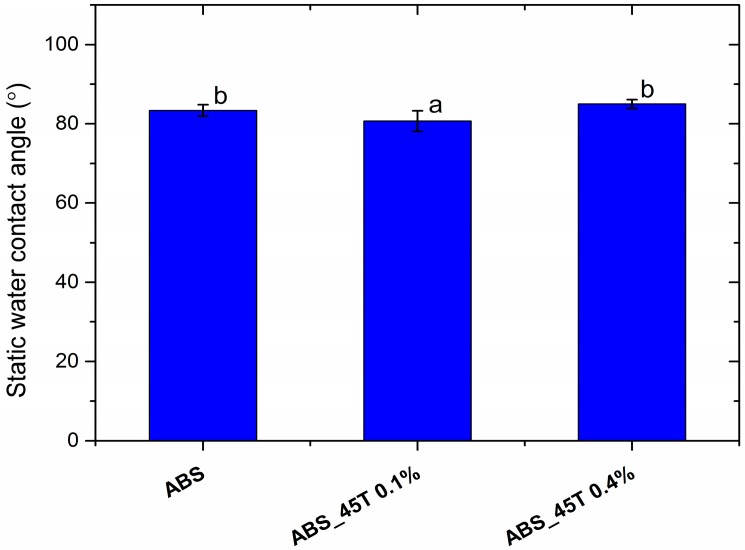
The static water contact angle of pure polymer and polymer containing 0.1% and 0.4% of silver nanoparticles AgNPs 45T by weight. Statistically significant differences (*p* < 0.05) between the tested materials are marked a–b.

The contact angle measurements established the characteristics of the sample surface. All polymer and composite materials containing silver nanoparticles are hydrophilic. The wettability angle measures 82° (Figure 3).

**Figure 4:**
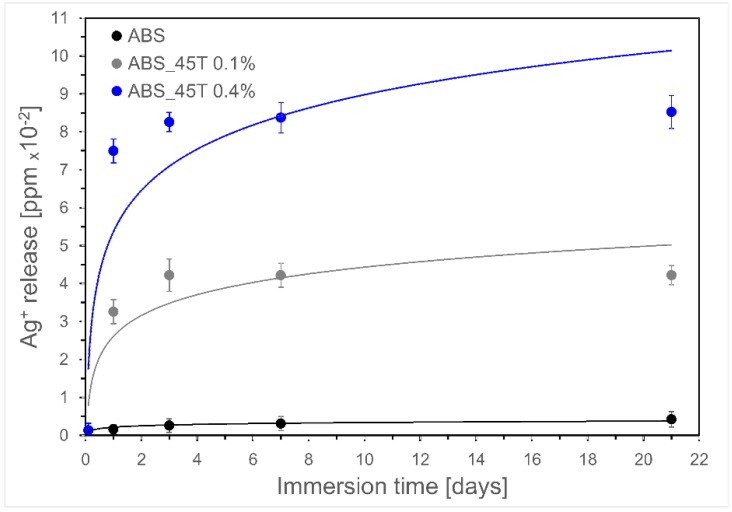
Kinetics of silver ions release during incubation in UHQ water up to 21 days.

The spectrometric analysis showed that the release of silver ions depends on the immersion time and the amount of nanosilver particles incorporated into the ABS matrix (Figure 4). The silver ions release increases as a function of time, however the highest increase was observed on the first day of incubation and is found to be marginal between the 3rd and 14th day. The reason for this observation can be explained by oxidation of silver ions in aqueous medium. Therefore, the rate of water diffusion in the composite sample is expected to govern the silver release [15]. During the incubation the silver ion release increased proportionally to the filler content.

Table 2 presents the results of antibacterial activity i.e. the number of bacteria that survived on the surface of polymer modified with silver nanoparticles and the control polymer without nanosilver. The tests have proved that the applied amount of 45T nanosilver—both 0.1% and 0.4% wt–eliminate the two species of bacteria. The obtained data indicates that AgNPs 45T resulting from green synthesis does not lose bactericidal efficacy when combined with the polymer. After 24 h-incubation, no bacterial growth was observed on the inoculated polymer discs. This was due to the largest dissolution of the silver ions in that period (Figure 4). The experiment was repeated with discs incubated for seven days in DMEM medium. The results showed weaker, but still significant, antimicrobial activity equal to three log bacterial growth reduction. This behaviour may be caused by a protein layer getting formed around the polymer discs, thus impeding the activity of the surface-bound silver. AgNPs are capable of killing both Gram-positive and Gram-negative bacteria and are effective against many drug-resistant microbes [2].

**Figure 5:**
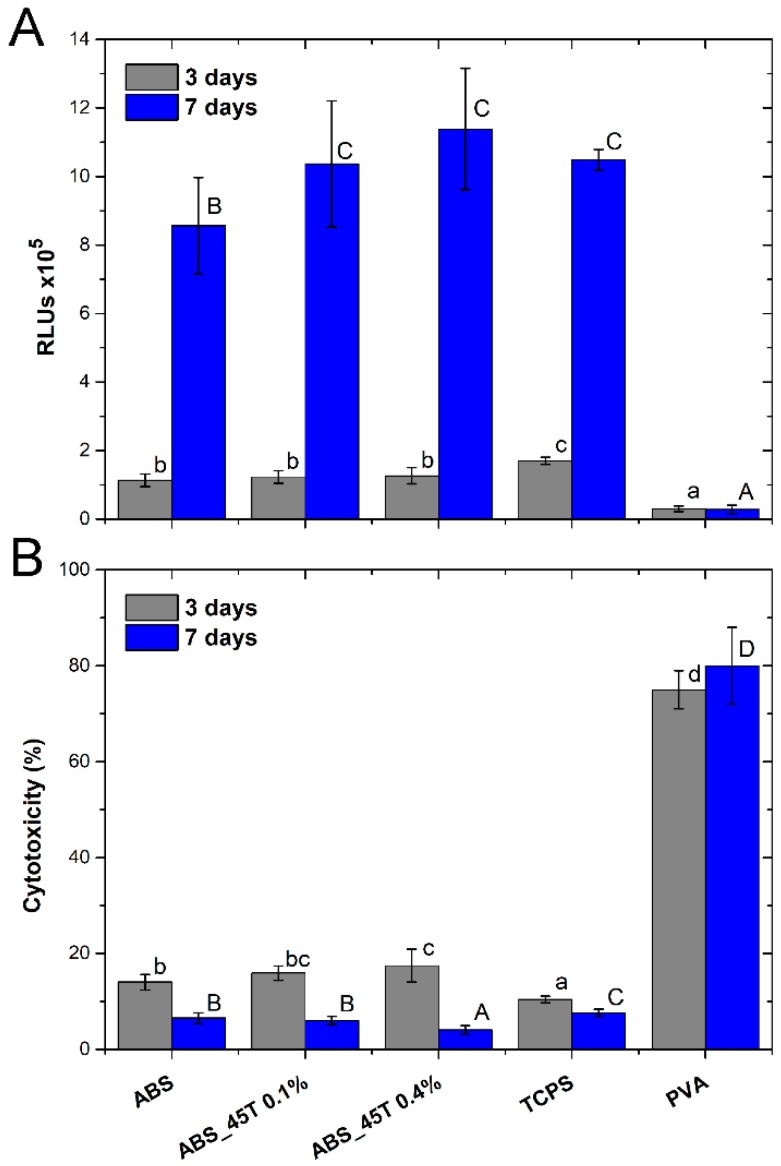
Relative number of cells (**A**) and cytotoxicity of materials (**B**) assessed in direct contact with Hs-680.Tr cells. The results are presented as mean values ± standard deviation. Statistically significant differences (*p* < 0.05) between the tested materials are marked a–c for 3-day culture and A–D for 7-day culture.

Figure 5A presents the relative number of Hs-680.Tr cells after 3 and 7 days of growth. A remarkable increase in the number of cells for all the materials after 7-day culture, as compared to the 3-day one, proves the high level of proliferation. Therefore, the conditions for fibroblast multiplication are advantageous.

On the third day of the experiment, the numbers of cells on the tested materials are similar, but the highest value is observed for the control TCPS. On the other hand, after 7 days the number of cells on the materials containing 0.1% and 0.4% AgNPs 45T is comparable to TCPS and the values are clearly higher than for the pure polymer.

Figure 5B presents cytotoxicity of the materials–the results established on the basis of ToxiLight assay detecting AK released from the damaged cells after 3 and 7 days of the culture. The merits are percentages of the damaged cells, as compared to their entire number. The research has proved that the tested materials show no cytotoxic effect on Hs-680.Tr fibroblasts.

After 3 days of the cell growth in direct contact with the materials, cytotoxicity increases along with the higher concentration of AgNPs 45T in the composites. Yet the mean value does not exceed 17.5%. The lowest cytotoxicity result (10.5%) is noted for the control TCPS.

After 7 days, the cytotoxicity significantly decreases for all the materials. The highest drop is observed for the material containing 0.4% AgNPs 45T, whose cytotoxicity was 4%. The pure polymer and ABS_45T 0.1% are characterized by similar values of about 6%. The cytotoxicity observed for materials is lowest than for the control TCPS.

**Figure 6:**
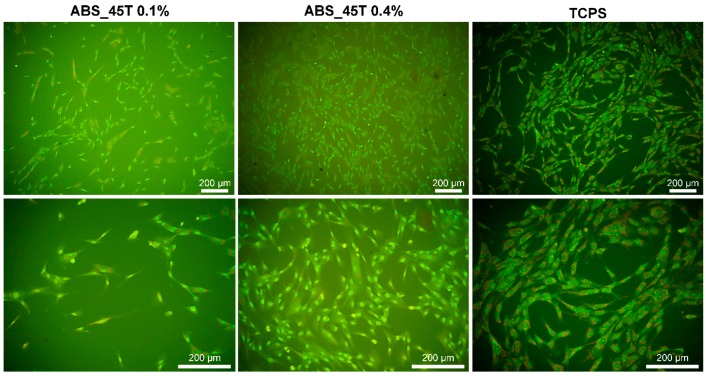
Hs-680.Tr fibroblasts adhered to TCPS and ABS samples containing 0.1% and 0.4% wt. of AgNPs 45T nanoparticles after 7-day culture.

After 7 days of culture, the fibroblasts have a proper shape and they grow evenly on the surface of both materials containing 0.1% and 0.4% AgNPs 45T. Such a conclusion is drawn on the grounds of the fluorescence microscopy images presented in Figure 6. Moreover, it is observed that a much higher number of fibroblasts appears on the material with the higher concentration of silver nanoparticles, which correlates with the results of the cell numbers displayed in Figure 5A. It seems that the cells in contact with ABS_45T 0.4% are better flattened than the ones on the 0.1% AgNPs 45T composite. Such results suggest that the presence of silver nanoparticles in the polymer matrix facilitates the fibroblasts adhesion and proliferation. This phenomenon may result from the increase in the surface roughness for the composites with higher nanoaddition (Figure 2).

## 3. Discussion

The biological activity of silver nanoparticles depends on many factors, such as the shape, size and synthesis precursors. The most popular shapes are spheres, double regular polyhedrons, triangles and cubes. The size of nanoparticles ranges from 1 to 100 nm in at least one dimension, depending on the method of synthesis.

There are three main types of synthesis: chemical, physical and biological [16]. The current trend in chemistry is creating “green” methods of synthesis that have the slightest impact on the environment. In most studies natural extracts are the reducing agents that limit the particle size. It means variability of the reaction mixture composition depending on a biological material.

By means of biological and chemical synthesis, despite the similar shape and size of AgNPs, completely different biological properties are obtained. This might result from various kinds of contamination. The chemical composition of nanoparticles directly affects the dissociation of Ag^+^ ions from their structure. Recently it has been proved that toxicity of AgNPs solutions results solely from silver ions [17]. It has been also reported that metallic silver can be oxidized to silver ions which show bactericidal efficacy. However, some works have revealed that only a small number of silver nanoparticles can be oxidized to silver and that most of them exist as silver nanoparticles [18].

The deposition of silver nanoparticles on an ABS surface by an ultrasound-assisted method was investigated. The ABS-silver nanocomposites prepared by an ultrasound method show significant antimicrobial activity [19]. However, silver nanoparticles were present only on the surfaces of polymer substrate and had a direct contact with microorganisms.

Our SEM outcomes showed that—thanks to the good dispersion of silver nanoparticles in the polymer matrix—the long-term bactericidal effect is sustained due to the gradual release of silver ions. The EDX analysis proved the even dispersion of silver ions in the whole volume. Additionally, the production method using injection moulding ensures a safe level of silver ions′ release. The bactericidal effect has been observed with concurrent lack of cytotoxic effect. Our experiments proved that even a small number of silver nanoparticles ensures the release of sufficient silver ions Ag^+^ ensuring bactericidal effect. The initial release of silver ions (for e.g., between days 1 and 3) must be from those silver particles which are encapsulated within the surface layers of the specimen. From the seventh day of immersion on, the silver ions release is slower and almost on a constant level, which might be responsible for the increasing biocompatibility after the AgNPs addition. Additionally, the increasing roughness of the sample surface leads to better cell adhesion. In the case of coatings, the rapid ions release results in a low biocompatibility of the material. What is more, the surfaces of implanted devices rapidly become coated with glycoproteins from tissue and plasma, with the possible exception of the inner surface of implant, and this has been cited as one of the main reasons for a clinical failure of silver-coated devices. The data presented by Furno et al. demonstrated that in the case of polymer impregnation there is both a depot effect and a diffusion pressure available to ‘push’ the silver ions through the conditioning film. In order to do this, there must be enough silver ions available over a sufficient period to exceed those lost to protein binding [20].

The long-term release performance can be considered an important factor from the viewpoint of practical applications. The subsequent release of silver ions results from the interior part of the specimen where water has to cross the diffusion barrier, which inhibits the oxidation process [21]. Conversely, in some cases, polymers may be porous enough to allow water to pass through the polymer and as a result, the silver nanoparticles can diffuse out of the polymer [18]. This mechanism may be responsible for the prolonged antibacterial efficiency. Some reports demonstrated the highest antibacterial activity due to the release of nanoparticles from the polymer promoted by LED irradiation. The light absorbed by polymer coating is transferred to heat, which induces the polymer chains motion and AgNPs elimination [22]. The increased antimicrobial activity of Ag-incorporated polymers may be increased by the high infiltration of the Ag component with a high bactericidal effect. Silver ions reportedly adhere to the negatively charged bacteria cell wall, changing the cell wall permeability. This action—coupled with the protein denaturation—induces cell lysis and death [23].

The studies confirm that the size of nanoparticles has a significant influence on the AgNPs activity. Silver nanoparticles measuring less than 10 nm display the highest bactericidal efficacy [24]. Due to the polymer/nanoparticles combination it is possible to obtain such a structure where only part of nanoparticles is active. Thus it is close to the effective measurements lower than 10 nm.

The increase in the number of eukaryotic cells noted for the composite may also result from the higher surface roughness as compared to the control during the AFM assessment. The application of nanoparticles stabilized by polymer during the synthesis prevents the dissociation of silver ions. The presented qualities of the composite lead to reducing its toxicity against eukaryotic cells and sustaining the bactericidal efficacy of AgNPs. This fact confirms the assumption concerning low cytotoxicity of the obtained composite which is similar to the one of the pure polymer.

The material surface is a vital issue in the case of contact with living organisms—the rougher the surface, the better bacterial colonization. The increased roughness also facilitates adhesion of osteoblasts to the material surface [25,26].

Higher roughness parameters promote adhesion of bacteria, microbial proliferation and formation of biofilms, which may lead to inflammatory processes, cell necrosis and even rejection of the implanted material [27]. In order to prevent such a disadvantageous course of action, the nanosilver modification is incorporated to achieve the bactericidal effect. In this way, the roughness parameter facilitates not only the bacterial colonization but also the antimicrobial efficacy. It is possible that the obtained roughness value encourages osteoblasts to adhere to the surface of the materials used for ear implants. For instance, in the case of prostheses for ossicular chain reconstruction, the roughness of the implant base promotes its fixation to the tympanic membrane. The balance between the bacteria and osteoblast adhesion must be achieved. A well-designed material endowed with slight nanometric roughness and controlled topography should prevent bacterial adhesion. Enriched with bactericidal agents, the material ought to inhibit microbial colonization and promote osteoblast adhesion and proliferation. However, such assumptions require further qualitative and quantitative studies assessing the relation between morphology of cells and bacteria and the material. There is a risk that too high roughness may lead to weaker contact of the cells and the substrate and thus the lower proliferation of cells [28,29].

On the grounds of the obtained results it may be concluded that acrylonitrile butadiene styrene (ABS) displays good wettability with distilled water. The modification with silver nanopowder (0.1–0.4% wt) does not affect the contact angle value and thus the surface characteristics. All the composite materials are characterized by the contact angle value similar to the one of pure polymer matrix.

The research proves that the tested ABS modified with silver nanoparticles AgNPs 45T is endowed with the bactericidal efficacy. Additionally, it is safe for eukaryotic cells. Therefore, it may be successfully used as a material for middle ear prostheses.

The results of bactericidal and cell culture tests showed that silver released from composites on one hand effectively imparts antibacterial properties, while on the other hand it does not show any cytotoxic effect on mammalian cells. Such a behavior confirmed the proper silver content was incorporated into the composites.

## 4. Materials and Methods

The commercially available ABS polymer material (poly)acrylonitrile butadiene styrene (INEOS SyrolutionEurope GmbH, Frankfurt, Germany) and composite materials modified with nanosilver (respectively 0.1% and 0.4% wt) were shaped as discs measuring 10 mm in diameter. Silver nanoparticles AgNPs 45T were obtained and tested at the Intercollegiate Faculty of Biotechnology, University of Gdansk and Medical University of Gdansk) according to Banasiuk et al. [30]. A water solution consisting of AgNO_3_ (4 mM), polyvinyl pyrrolidone (200 mg per 100 mL) and sodium hypochlorite, NaClO (0.5 mL of 5.25% solution per 100 mL) was irradiated in a borosilicate glass for 10 min, using a 420 nm LED light source. The colour of the solution changed from white opaque to purple, showing the synthesis of the nanoparticles. After the synthesis, the nanoparticles were centrifuged. The remaining solvent was evaporated. The size and shape of nanoparticles was estimated, basing on SEM and TEM described in our previous work [30]. The silver nanoparticles were spherical and measured below 50 nm in diameter.

**Figure 7:**
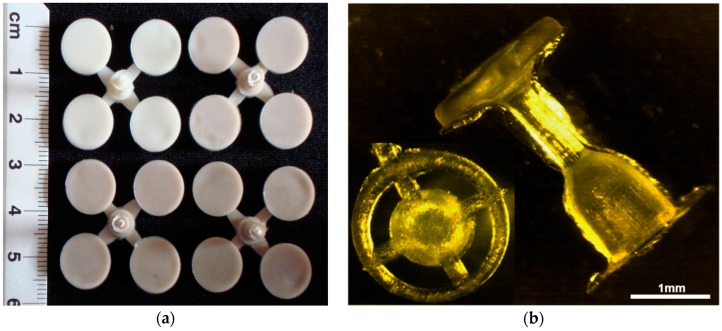
(**a**) Samples for in vitro tests; (**b**) middle ear prostheses.

The method of obtaining the implant prototypes was based on the previous studies, i.e., the production of samples for mechanical and biological testing. The procedure of obtaining the prostheses consisted of a few steps. First, the granulate was prepared and dried in the laboratory dryer at 80°C for 6 hours. Next, the nanosilver particles were incorporated and homogenized with polymer granules in the plasticizing chamber using a 0.8 m length screw. Subsequently, the material was injected into the steel moulding form, cooled and extracted. The injection parameters were selected and adapted for the process according to characteristic data sheet of polymer manufacturer (injection temperature in three zones 240 °C, injection pressure 80 kg·cm^−2^, flow 70%). The disc-shaped samples and middle ear prostheses obtained through injection moulding and extrusion are presented in Figure 7.

Then, the sterilization process was run by means of the low-temperature plasma (Sterrad 120 apparatus, Johnson & Johnson Medical Ltd., Wokingham, UK) using H_2_O_2_ in DUO Cycle (2 × 45 min). Biological tests were conducted on the sterilized materials to assess their bactericidal efficacy, cytotoxicity and cell proliferation.

### 4.1. Experimental

#### 4.1.1. SEM

An Nova NanoSEM 200 scanning electron microscope (FEI Netherlands, Eindhoven, The Netherlands) coupled with an Genesis XM X-ray microanalysis system (EDAX, Tilburg, The Netherlands) featuring the EDAX Sapphire Si(Li) EDX detector was used to perform a detailed examination of the microstructure of the produced materials. The measurements and observations were conducted in high vacuum conditions, with back scatter electron detector (BSE), the accelerated voltage was 10–18 kV. The samples were coated with a carbon layer.

#### 4.1.2. AFM

The topographical evaluation of the ABS and ABS_45T composites was performed via Atomic Force Microscope (AFM, MultiMode 8 microscope, Bruker, Karlsruhe, Germany), using antimony-doped silicon tips (spring constant = 40N/m), operating in a tapping mode.

#### 4.1.3. Surface Wettability

The surface wettability was evaluated by static water contact angle measurements. The contact angle was determined by the sessile drop method with an automatic drop shape analysis system DSA 10 Mk2 (Kruss GmbH, Hamburg, Germany). UHQ-water droplets of 0.25 μL were applied on each pure and dry sample. The experiments were carried out in constant conditions (temperature and humidity). The apparent contact angle was calculated as an average of 10 measurements and expressed as a mean ± standard deviation (SD).

#### 4.1.4. ICP-MS

The ABS and ABS_45T composites samples were incubated at 37 °C in 50 mL of UHQ water for 21 days. The in vitro release of silver ions was studied by means of Inductively Coupled Plasma Mass Spectrometry (ICP-MS), using an ICP-MS Plasma 6100 spectrometer (Perkin-Elmer, Waltham, MA, USA). In order to protect silver ions (Ag^+^) from reduction into metallic silver and prior to performing ICP-MS analysis, the filtered samples were acidified with nitric acid, up to the final concentration of 0.1 mol/L. The silver concentrations in the investigated samples were determined using ICP-MS at *m*/*z* 107, with the external standard calibration procedure.

#### 4.1.5. In Vitro Bactericidal Efficacy Tests

**Figure 8:**
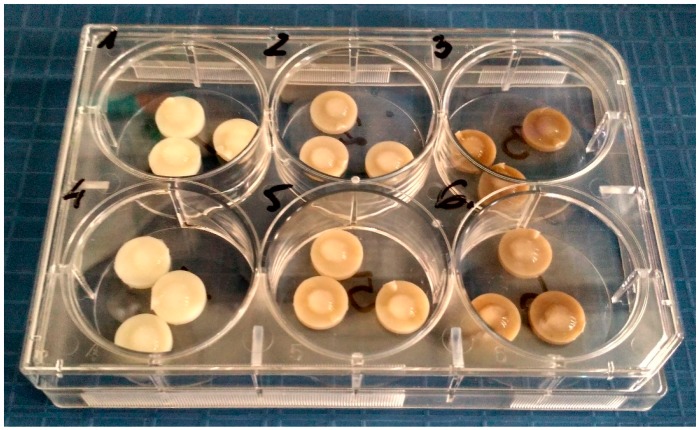
Exemplary 6-well plate with discs (control—well 1 and 4; AgNPs 45T 0.1%—well 2 and 5; AgNPs 45T 0.4%—well 3 and 6) topped with the bacteria/soft-top agar suspension.

The two types of polymer discs containing AgNPs 45T silver nanoparticles (0.1% and 0.4%) were tested to establish the bactericidal efficacy, using the modified method according to the ASTM E 2180-01 norm (*Standard Test Method for Determining the Activity of Incorporated Antimicrobial Agent(s) In Polymeric or Hydrophobic Materials*). The only alteration was introduced to match the norm with the size of polymer samples. Every sample was tested according to the following procedure. First, bacterial suspensions were prepared (1.5*10^5 CFU/mL) [CFU − colony forming unit − a measure of viable bacterial cells]: *Staphylococcus aureus* ATCC 19660 and *Pseudomonas aeruginosa* PAK [31] Next, 100 mL of 0.3% agar solution and 0.85% NaCl (soft-top agar) solution were prepared for each pathogen and 1 mL of the bacterial suspension was added to soft-top agar solutions respectively. The three samples after manufacturing and after 7-day incubation in DMEM (ATCC, Manassas, VA, USA) containing 10%FBS (HyClone, Logan, UT, USA) were placed in 6-well cell culture plates (wells of 3.5 cm-diameter). One type of bacteria was applied into one well. The samples did not come in any physical contact in the course of the experiment (Figure 8). 50 μL of soft-top agar bacterial suspension was placed on the samples that were incubated at 37 °C for 24 h. After 24 h the polymer samples were transferred to 5 mL-test tubes containing 2 mL of Brain Heart Infusion (BHI) broth. The test tubes were treated with ultrasound for 1 min at room temperature and vortexed (1 min) to transfer bacteria to the broth. At the next stage 400 μL of the BHI-bacteria suspension was mixed with 600 μL of straight BHI. Subsequently, 100 μL was planted on the BHI-soft-top agar composition and incubated at 37 °C for 24 h. After the incubation the bacteria colonies were counted.

#### 4.1.6. In Vitro Cell Tests

The commercially available Hs680.Tr (human fibroblast trachea cells) line was chosen in order to examine the viability/proliferation of cells in direct contact with the materials dedicated for the middle ear implant and their cytotoxicity. The type of the selected cells was consulted and approved by ear, nose and throat specialists from Otolaryngology Clinic UJ. The cell lines came from the American Type Culture Collection (ATCC). The studies were conducted according to the norm PN-EN ISO 10993-5 “Biological evaluation of medical devices. Tests for in vitro cytotoxicity”.

The Hs680.Tr fibroblasts were cultured in polystyrene 75 cm^2^ flasks (Nunclon, Thermo Fisher Scientific, Waltham, MA, USA) in DMEM medium (ATCC, Manassas, VA, USA) with 10% addition of fetal bovine serum (HyClone, USA). The cell culture was conducted in HeraCell incubator (Thermo Scientific, Darmstadt, Germany) in 5% CO_2_ atmosphere at 37 °C. The Hs680.Tr cells from the third passage were used for tests. The cell solution was obtained by the double PBS wash and addition of 5% trypsin-EDTA (HyClone). Having been rinsed and revolved, the cells were placed in fresh medium to obtain density of 2 × 10^4^ cells/mL. Then, 0.5 mL of the cell suspension was placed in wells of 48-well plates (Nunc) containing sterile discs of the examined materials and 0.5 mL of medium. The control was tissue culture polystyrene (TCPS). TCPS and PVA served as the negative and positive control for the cytotoxicity tests.

The Hs680.Tr cell culture in the presence of the tested samples was conducted for 3 and 7 days. After the set time supernatant from above the cell culture was transferred onto Optiplate microplates (PerkinElmer, Waltham, MA, USA) to establish the cytotoxicity of the materials, whereas the cells growing on the samples′ surface underwent viability/proliferation tests.

The fibroblast cytotoxicity assay of the materials was conducted via bioluminescence method, using Toxilight Bioassay Kit (Lonza, Walkersville, MD, USA) that measures the release of adenylate kinase (AK) from damaged cells. AK is a robust protein present in all eukaryotic cells, which is released into the culture medium when cells die. The luminescence was measured with PolarStar Omega plate reader spectrophotometer (BMG Labtech, Ortenberg, Germany). The test was conducted on the supernatant from the cell culture and the results were compared to the entire enzyme concentration (proportional to the entire number of cells) released from all cells.

ToxiLight 100% Lysis Reagent set (Lonza) was used to disintegrate the cytomembrane of the cultured cells. Then ToxiLight Bioassay Kit was used in order to establish the entire number of cells confirming their viability and proliferation. The amount of the released adenylate kinase was assessed with PolarStar Omega reader (BMG Labtech, Ortenberg, Germany).

Two samples of each series were used for morphological observation under the fluorescence optical microscope. The cells adhering to the materials were being dyed for 1 min with acridine orange AO solution (Sigma-Aldrich, Saint Louis, MO, USA). After washing with PBS (HyClone), the cells were examined by means of an Olympus CX-41 microscope (Olympus, Japan) equipped with a fluorescence device. A digital camera E-520 (Olympus) was used to take photographs of the cells.

#### 4.1.7. Statistical Analysis

The results were analyzed using one-way analysis of variance (ANOVA) with Duncan post hoc tests which were performed with Statistica 10 (StatSoft^®^, Tulsa, OK, USA) software. The results were considered statistically significant when *p* < 0.05.

## 5. Conclusions

The early experiences with the acrylonitrile butadiene styrene (ABS, INEOS Syrolution Company) modified with silver nanoparticles AgNPs 45T have shown encouraging results. The composites are found to release silver ions at a concentration level capable of rendering antimicrobial efficacy even with the lowest concentration of silver nanoparticles in the material. The conclusions are promising as the material has proved to be biocompatible, thus it is suitable for middle ear implants used in ossicular chain reconstruction.

## Figures and Tables

**Table 1:** The content of elements in polymer and ABS_45T composites measured with EDX.

Weight Fraction	wt. %			
Material	C K	N K	O K	Ag L
ABS	95.65	1.39	2.95	-
ABS_45T 0.1%	94.77	1.85	3.25	0.14
ABS_45T 0.4%	89.69	2.82	7.13	0.35

**Table 2:** Antimicrobial efficacy of polymer composites before and after 7-day polymer incubation in DMEM medium containing 10% FBS.

Bacteria Species	*Staphylococcus aureus* *Gram-positive*		*Pseudomonas aeruginosa* *Gram-negative*	
Bacteria Inhibition	*% of growth inhibition*			
Material	0 days	7 days	0 days	7 days
ABS	0	0	0	0
ABS_45T 0.1%	100	98.2	100	99.4
ABS_45T 0.4%	100	99.7	100	99.8

## Abbreviations

ABS	acrylonitrile butadiene styrene
AgNPs	silver nanoparticles
AK	adenylate kinase
TCPs	control sample
Hs680.Tr	human fibroblast trachea cells
